# Therapeutic Fasting in Enteric Fever

**Published:** 1904-12-17

**Authors:** 


### THERAPEUTIC FASTING IN ENTERIC FEVER.

A suggestion has recently been put forward by Dr. Harbin[1] to the effect that in certain cases of enteric fever the patient may with considerable advantage be deprived for a short while of all nourishment by the mouth. He points out that cases attended with diarrhœa usually have a greater degree of toxæmia than cases in which there is constipation. Emaciation, he adds, occurs independently of the amount of food taken into the stomach, and this suggests that little food is assimilated while the remainder furnishes a good medium in which the typhoid bacilli can grow and produce large quantities of toxin. Therefore, in robust adults, who are just those patients who are most liable to succumb from enteric fever, he recommends restricted diet and fasting as the most important field of therapy, as the dangers from inanition in these cases are much less than the dangers from toxæmia. When the patient first comes under treatment Dr. Harbin recommends that he should be deprived of all food for a period of one to three days, and at the end of that time he should be given a very restricted diet consisting of broths, oysters, cocoa, egg albumin and so forth in prescribed amounts. Milk should be absolutely forbidden. Gelatin is valuable both as a food and as a prophylactic against hæmorrhage, and it should be used as a routine; a teaspoonful is the usual dose, the gelatin being dissolved in a little hot water and added to the other foods. Hydro-therapy should also be fully employed when necessary. Dr. Harbin states that, working on these lines, he has had only four deaths among 116 consecutive cases of typhoid, and only two deaths among the 113 white patients included in the series. Unfortunately he omits to mention how many of the 116 cases were of a sthenic character and were treated by fasting and restricted diet. Nevertheless his suggestion is one which it is well worth while meditating upon, and perforce attempting in practice.

[1] Med. Rec., Nov. 12.

